# Reconstruction of visible light optical coherence tomography images retrieved from discontinuous spectral data using a conditional generative adversarial network

**DOI:** 10.1364/BOE.435124

**Published:** 2021-10-07

**Authors:** Antonia Lichtenegger, Matthias Salas, Alexander Sing, Marcus Duelk, Roxane Licandro, Johanna Gesperger, Bernhard Baumann, Wolfgang Drexler, Rainer A. Leitgeb

**Affiliations:** 1 Center for Medical Physics and Biomedical Engineering, Medical University of Vienna, Austria; 2 Christian Doppler Laboratory for Innovative Optical Imaging and Its Translation to Medicine, Medical University of Vienna, Austria; 3 EXALOS AG, Switzerland; 4 Department of and Biomedical Imaging and Image-guided Therapy, Computational Imaging Research, Medical University of Vienna, Austria; 5 Institute of Visual Computing and Human-Centered Technology, Computer Vision Lab, TU Wien, Austria; 6 Division of Neuropathology and Neurochemistry, Department of Neurology, Medical University of Vienna, Austria; 7 These authors contributed equally

## Abstract

Achieving high resolution in optical coherence tomography typically requires the continuous extension of the spectral bandwidth of the light source. This work demonstrates an alternative approach: combining two discrete spectral windows located in the visible spectrum with a trained conditional generative adversarial network (cGAN) to reconstruct a high-resolution image equivalent to that generated using a continuous spectral band. The cGAN was trained using OCT image pairs acquired with the continuous and discontinuous visible range spectra to learn the relation between low- and high-resolution data. The reconstruction performance was tested using 6000 B-scans of a layered phantom, micro-beads and ex-vivo mouse ear tissue. The resultant cGAN-generated images demonstrate an image quality and axial resolution which approaches that of the high-resolution system.

## 1. Introduction

Optical coherence tomography (OCT) is an interferometric technique, where the image contrast is based on the back-scattered and reflected light of the sample morphology. Using a low-coherent light source, three dimensional (3D) cross-sectional images are retrieved non-invasively [1]. Over the last decades OCT has become an important diagnostic tool, especially for ophthalmology [2]. Furthermore, OCT is increasingly recognized in other diagnostic fields such as neuro-, skin and endoscopic imaging [3–5].

In OCT, the axial resolution is determined by the spectrum of the used light source. The broader the spectrum and the lower the chosen central wavelength, the higher the axial resolution can get [6–8]. The development of supercontinuum laser sources has pushed the axial resolution limits for OCT down to sub-micron imaging [6,9]. In this context, especially visible-light OCT (vis-OCT) has shown to be valuable in a broad range of ex-vivo and in-vivo applications [7–10]. Vis-OCT has been utilized to investigate structures in the in-vivo murine and human eye as well as in ex-vivo brain tissue samples with sub-micrometer axial resolution [7–11]. Supercontinuum laser sources can provide spectral ranges from 425 to 2350 nm and therefore extremely high resolution possibilities [12]. However, these sources are cost intense and typically have a rather high relative intensity noise (RIN) [13]. Traditionally, light sources working in the near-infrared region have been used to perform OCT such as at wavelengths of 800 , 1000 , 1300 or 1500 nm [14,15]. The realization of non supercontinuum sources with broad spectral ranges is still a technological challenge [16]. For example for superluminescent diodes (SLDs), one option is to combine multiple semiconductors, where each is working in a certain wavelength region, to increase the spectral range [17–19]. Generating such light sources has been proven to be beneficial for OCT imaging, however, no spectral gaps should be introduced in this process. If a spectrum with gaps is used to perform OCT imaging, the axial resolution is reduced and sidelobe artifacts are introduced, which in turn degrades the image quality [20]. As an alternative approach, research has been conducted to overcome these spectral gaps using numerical methods [21,22].

Deep-Learning can substitute algorithms and human inputs for many different applications [23]. This approach has substantial impact in many different fields, such as ophthalmology, where classification and image recognition are needed [24,25]. Generative Adversarial Networks (GANs) are deep learning algorithms used for image generation. Just to mention one promising application, the so called MedGAN network is used to perform medical image-to-image translation in magnetic resonance imaging data [26]. Conditional GANs (cGAN) are a modification of classical GANs where the input is not random noise but also an image [27]. In OCT deep learning has widely been applied, for example for automatic diagnosis or segmentation, anomaly detection and denoising of image data [28–34]. Further, in ophthalmology neuronal networks have been trained to generate synthetic OCT or OCT angiography data [35,36]. Recently, deep learning strategies such as GANs have also been used to generate high resolution out of lower resolution OCT images [37–42]. However, the presented studies so far, using deep learning approaches such as GANs in combination with OCT data to improve the image quality and resolution, were all performed on continuous spectral data. Furthermore, the low resolution OCT data were generated by downgrading the original high resolution once. This has the advantage that the low and high resolution images are perfectly matched, but lacks a practical application when using real OCT setups generating low resolution tomograms.

In this work, based on a cGAN network we reconstruct for the first time OCT images taken with a discontinuous spectrum, generated by two SLDs to retrieve images acquired with a broadband visible-light source. The network is trained and tested with tomograms obtained with a source showing a spectral gap and with a broad-band supercontinuum source. In this work the complex OCT data, so not only the amplitude but also the phase are used as an input to train the proposed network. Data acquired from phantoms and ex-vivo mouse ear tissue are presented. The results show an improvement in axial resolution and image quality after utilizing the presented cGAN.

## 2. Methods

### 2.1 Data acquisition

A visible light optical coherence microscopy (OCM) setup was used to acquire the training data. A detailed description of the setup can be found in Lichtenegger et al. [8,43]. A supercontinuum light source (NKT Photonics SuperK EXTREME EXU-6) in combination with a filter box (NKT Photonics VARIA) provided a broad, visible spectrum for imaging (425-685 nm). The resulting measured axial resolution in air was 1.2 μ m. A 20 × commercial objective lens (Olympus, UPLFLN 20XP) was used for the acquisitions (measured transverse resolution 2.0 μ m) providing a field-of-view of 400 μ m × 400 μ m. Data sets comprised 500 × 500 × 4096 pixels and were acquired in 8.3 seconds. The power at the sample was measured to be 0.8 mW. The post-processing steps for the OCT data sets can be found in Lichtenegger et al. [43].

**Fig. 1.**
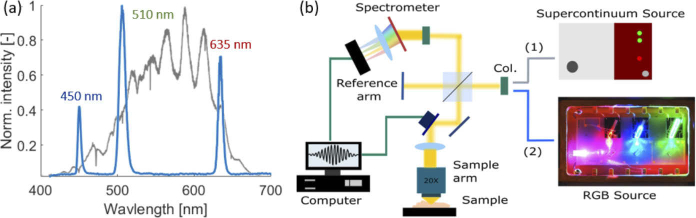
The EXALOS RGB SLED source. (a) The discontinuous spectrum of the light source, with the three peaks located at 450 nm (blue), 510 nm (green) and 635 nm (red). The peak heights were adjusted to follow the broadband visible light spectrum. Additionally, the spectrum of the supercontinuum source is indicated in grey in the background. (b) A sketch of the setup showing the two sources used. A photograph of the open 14-pin Butterfly package (25 mm x 12 mm) is shown. (Col. = Collimator)

For imaging with the discontinuous spectrum, an RGB (Red-Green-Blue) superluminescent light emitting diode SLED source (EXC250010 on EBD9200 driver board) with a polarization maintaining (PM) output fiber from EXALOS [44] was used in the exact same OCT setup. To switch between the two light sources simply the input fiber had to be exchanged, see Fig. 1(b). Additionally, a Glan-Thompson polarizer was inserted in front of the fiber leading to the spectrometer to reduce unwanted cross-correlation artifacts. Figure 1 shows the discontinuous spectrum and an image of an open 14-pin Butterfly package of the EXALOS light source is shown in the sketch of Fig. 1(b).

**Fig. 2.**
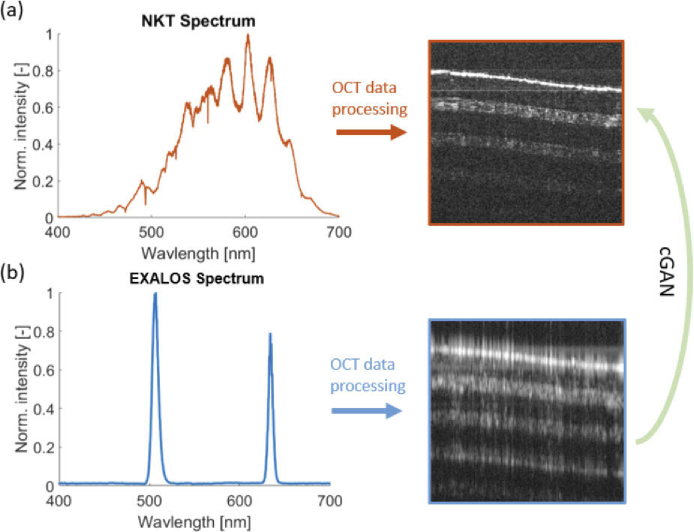
The concept of the conditional generative adversarial network approach (cGAN). The cGAN network was trained to retrieve high resolution OCT images with a quality comparable to an NKT source acquisition (a) from OCT data generated by the discontinuous RGB EXALOS source (b).

The source delivers three distinct peaks located in the blue ( $\lambda_{c}$ =450 nm, Δλ = 5 nm), green ( $\lambda_{c}$ =510 nm, Δλ = 10 nm) and red ( $\lambda_{c}$ =635 nm, Δλ = 6 nm) spectral range. The maximal output power of the three wavelength regions was 6.9 mW (blue), 5.2 mW (green) and 5.8 mW (red), respectively. However, for imaging the overall power at the sample was kept at 0.8 mW comparable to the one of the NKT source. The power of the visible-light spectrum of the original OCM setup was rather low in the blue wavelength region . The power in the blue region was decreased by 83% compared by the average output power per wavelength region. and so was the quantum efficiency of the camera (lower then 30%), which is why the blue peak was not used in the following experiments, see Fig. 2(b).

First, scotch tape and a micro bead phantom were imaged, and further ex-vivo ear tissue of a mouse was investigated. The micro bead phantom comprised iron oxide particles (0.01 %, particle size ranged from 20 - 100 nm) embedded in resin. The used mouse ear tissue was collected from a control wild type mouse with a B6SJL background and was fixated using 4 percent paraformaldehyde. Animal experiments were approved by the local ethics committee and by the Austrian Federal Ministry of Education, Science and Research under protocol BMBWF-66.009/0279-WF/V/3b/2018. Immediately after the OCT measurements, the ex-vivo mouse ear sample was processed for histologic workup. Hematoxylin and eosin (H&E) staining was performed and digital micrographs were acquired with a slide scanner (C9600-12, Hamamatsu).

### 2.2 Conditional adversarial network

The network architecture proposed was inspired by the Pix2Pix Network from Isola et al. [45] and has been adapted for the high-resolution reconstruction of OCT scans in the following way: The input of the network proposed consisted of single discontinuous OCT B-scans and was trained to generate high-resolution OCT B-scans. The general idea of the approach presented is illustrated in Fig. 2. The original broadband spectrum is shown in Fig. 2(a) and the discontinuous one in Fig. 2(b). The main modification to the original Pix2Pix Network was to input phase and amplitude data and further optimize the loss function, as described in details in following section.

#### 2.2.1 Architecture

**Fig. 3.**
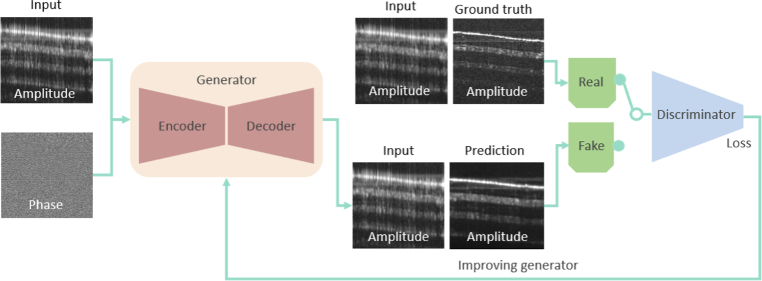
The conditional generative adversarial network (cGAN) architecture. The generator gets the amplitude and phase as an input to predict the high resolution B-scan image. The discriminator uses the predicted high-resolution images and the ground truth images from the full spectrum source to determine if the provided input is a real high resolution or a reconstructed image by the generator.

The Deep-Learning algorithm proposed is based on a cGAN consisting of 2 components: the Generator G and the Discriminator D . The architecture of the network is shown in Fig. 3.

**Fig. 4.**
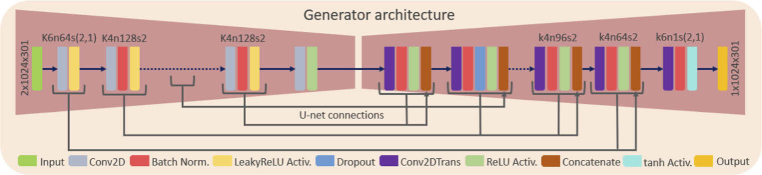
The architecture of the generator (Two dimensional convolution (Conv2D), Leaky rectified linear activation (LeakyReLU Activ.), Two-dimensional transposed convolution (Conv2DTrans), Rectified linear activation (ReLU Activ.), Tangens hyperbolicus activation (tanh Activ.)).

The Generator ( G ) was implemented as a cascade of an encoding and a decoding path with skip connections between them (cf. Fig. 4 for a detailed illustration of the generator’s architecture). G was trained to produce a high-dimensional B-scan $y_{recon}$ based on its multi-channel input x , which was formed by a B-scan’s amplitude and phase acquired with a discontinuous source. The discriminator’s input $x_{D}$ comprised either a generated image by the generator $y_{recon}$ or a real high-resolution B-scan $y_{real}$ and its task was to decide if $x_{D}$ was a generated or a real image. The training of the generator and discriminator was performed simultaneously. The discriminator focused on maximizing the probability of assigning the correct label to generated and real images. The focus of the generator lied in fooling the discriminator by learning a model distribution $P_{m}$ in a lower dimensional latent space Z from the data distribution $P_{data}$ and consequently improving the generation of realistic looking high-resolution reconstructions.

The objective function O of the cGAN (cf. Eq. 1), is based on the formulation proposed in [45] and was extended with the perceptual loss introduced in C. Wang et al. [46].

(1) $$O=arg\;\min_{G}\max_{D}L_{cGAN}(G,D)$$

O consisted of a generator loss and a discriminator loss term, introduced in Eq. (5) and (2) respectively. The discriminator loss term $L_{D}$ was comprised by the perceptual adversarial loss $L_{P}$ term and two binary cross entropy (BCE) loss terms ( $L_{x_{D}=real}$ , $L_{x_{D}=recon}$ ).

(2) $$L_{D}=\theta_{D}\frac{1}{2}(L_{x_{D}=real}+L_{x_{D}=recon})+max(0,(m-(L_{P})))$$

The perceptual adversarial loss $L_{P}$ (cf. Eq. (3), [46]) was defined as the sum of L1 distances between the reconstructed $y_{recon}$ and the real images $y^{real}$ observed at the different layers $d_{j}$ of the discriminator, weighted by $\lambda_{j}$ ( j was the hidden layer, F the number of hidden layers, $d_{j}(.)$ the image representation on the $j^{th}$ hidden layer, N the number of training samples and m a threshold).

(3) $$L_{P}=\sum_{j=1}^{F}\lambda_{j}(\frac{1}{N}\sum_{i=1}^{N}\|d_{j}(y_{real}(i))-d_{j}(y_{recon}(i))\|)$$

Note that ||⋯|| denotes the L1 norm. Beside the perceptual adversarial loss, a BCE loss (Eq. 4)) is computed separately for real and generated images between the predicted discriminator label $\ell^{pred}$ and the discriminator’s input true label $\ell^{true}$ .

(4) $$L_{x_{D}}=\frac{1}{N}\sum_{i=1}^{N}(\ell_{i}^{true}\log\ell_{i}^{pred})+(1-\ell_{i}^{true})\log(1-\ell_{i}^{pred})$$

The generator loss $L_{G}$ is defined in Eq. 5) and is formed by the sum of a BCE loss ( $L_{x_{D}=recon}$ ), estimated based on the true labels and predicted labels by the discriminator of generated images, and a weighted L1 loss term.

(5) $$L_{G}=\theta_{G}L_{x_{D}=recon}+\lambda_{G}(\frac{1}{N}\sum_{i=1}^{N}\|y_{real}(i)-y_{recon}(i)\|)$$

θ is the hyper-parameter term, which balances the influence of generative adversarial loss and perceptual loss. The code for the network can be found in the link provided https://github.com/AlexanderSing/OCT-cGAN.

### 2.3 Experimental Setup

The input of the generator consisted of OCT B-scans acquired as described before. The discriminator was fed with the full spectral OCM B-scan data and the discontinuous B-scan images. As an input, the phase and the amplitude were always passed to the generator. The phase data were extracted as angle maps from the complex OCT signal and were normalized between 0 and 1. Both the generator and the discriminator were updated by minimizing the loss functions using the Adam optimizer. For evaluation, following Yang et al. [47], the Fréchet Inception Distance [48] and image quality measures (structural similarity index (SSIM) [49] and peak signal-to-noise ratio (PSNR) [50]) were used. For the loss computation the loss function parameter $\lambda_{G}$ was set to 100 , $\theta_{G}$ to 1, $\theta_{D}$ to −1 , m to 50 and $\lambda_{j}$ to ( 5.0 , 1.5 , 1.5 , 1.5 , 1.0 ). An Adam optimizer was used to minimize the loss function with a learning rate set to 0.0002 and β values to 0.5 and 0.999 respectively. Additionally, the training made use of an image pool, which kept a set of images, that were already used for training during one epoch. For every generator training step, this image pool was used for training the discriminator multiple times. The image pool was first filled up with images until its maximum size was reached and afterwards there was a 50 % chance that a new training image replaced one of the ones in the pool. For the cGAN training the pool size was set to 50 and the discriminator was trained three times per generator step. Additionally, batch normalization was used with a batch size of 1 .

#### 2.3.1 Training-, validation- and testdata setup

For the cGAN, 6000 distinguished B-scan images were acquired in total, 2000 from scotch tape, 2000 from micro beads and 2000 from the ex-vivo mouse ear tissue. Additionally, 3000 data (1000 from each sample) sets were generated by a synthetically generated discontinuous spectrum. The synthetically discontinuous data were used to support the training of the network. To generate those data sets, the original broadband NKT spectrum was multiplied in post-processing with a combination of two Gaussian peaks located at 510 nm with a bandwidth of 10 nm and at 635 nm with a bandwidth of 6 nm. The synthetically gapped OCT data were also processed as described in Lichtenegger et al. [43]. For the training, each time 1500 images of the scotch tape and the micro-beads and 1000 synthetically generated data of the mouse ear were used. This combination of training data sets turned out to yield the best results. The training data was strictly separated from the test sets, which consisted of 500 images per object type. Furthermore, the test and training data were acquired on distinct locations of the investigated samples. At the beginning of the training, the training data was randomly split into an actual train and a validation set with a 95:5 split. During the training, the order of the images in the training set was shuffled; data augmentation for each epoch and image was performed with a probability of 0.5.

#### 2.3.2 Hardware

The deep learning algorithm was implemented using PyTorch [51] & PyTorch Lightning [52] on a machine with an AMD Ryzen 3950X, 64 GB RAM (3600 MHz DDR4 Dual-Channel) and an NVIDIA RTX 3090 with 24 GB VRAM. Additionally, the training was monitored using TensorBoard (Version 2.2.1) [53]. Training the network averaged about 20 minutes per epoch while inference took about 30 ms per image, averaged over 500 images. The total run-time of the training was 10 hours.

## 3. Results

The results presented are structured according to the data type evaluated: (A) scotch tape images (B) micro-beads images and (C) ex-vivo mouse ear images. In the following, the "Input" data are the B-scans acquired with the discontinuous source, the "Ground Truth" data were acquired with the full spectrum and the "Prediction" was generated by the proposed cGAN. In the last results section the quantitative evaluation of all data types is presented. All results presented are generated from data which the network has never seen before.

### 3.1 Scotch tape image data

**Fig. 5.**
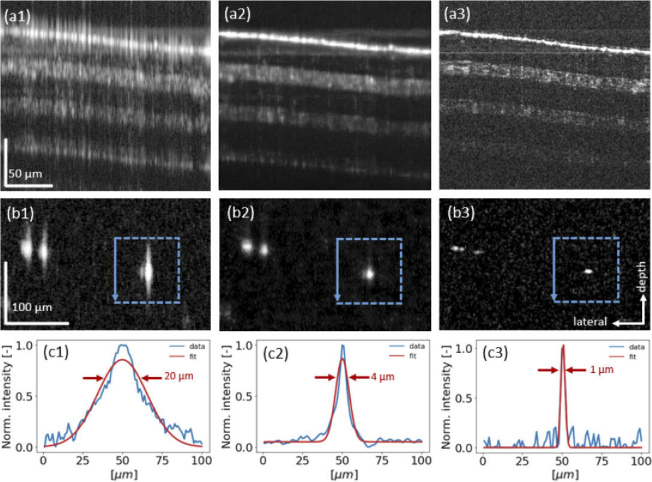
Results of the scotch tape (a1)-(a3) and a zoom-in into a region of interest in the micro-bead data (b1)-(b3). (a1) Input B-scan data. (a2) The prediction B-scan data. (a3) The ground truth B-scan data. (b1) Input B-scan data. (b2) The prediction B-scan data. (b3) The ground truth B-scan data. (c1)-(c3) show the axial profile plots with the respective Gaussian fits in the micro-bead marked with the blue dashed squares in (b1)-(b3), respectively. The arrow in the left side of the blue box, indicates that the profile of the micro-bead was evaluated along the depth.

The results of the scotch tape imaging, namely the input, the predicted high-resolution reconstruction and the ground truth B-scan data are shown in Fig. 5(a1)-(a3), respectively. It can be observed that the cGAN drastically reduces image artifacts, introduced by the spectral gaps and increases the axial resolution.

### 3.2 Micro-beads image data

To verify the improvement of the axial resolution, micro-beads were imaged. The input, the prediction and the ground truth B-scan data, in a zoomed-in region of interest, for the micro-bead measurements are shown in Fig. 5(b1)-(b3), respectively. It can be observed that the cGAN is able to improve the axial resolution, see Fig. 5(b1) compared to Fig. 5(b2). Profile plots in combination with a Gaussian fit were used to evaluate the full-with-at-half-maximum of the peaks in depth (z-direction) of selected micro-beads marked by blue squares are shown in Fig. 5(c1)-(c3), respectively. Data are shown in log scale. The cGan was able to improve the axial resolution in the OCT images generated by the the discontinuous spectrum and by a factor of 5.

### 3.3 Ex-vivo mouse ear image data

**Fig. 6.**
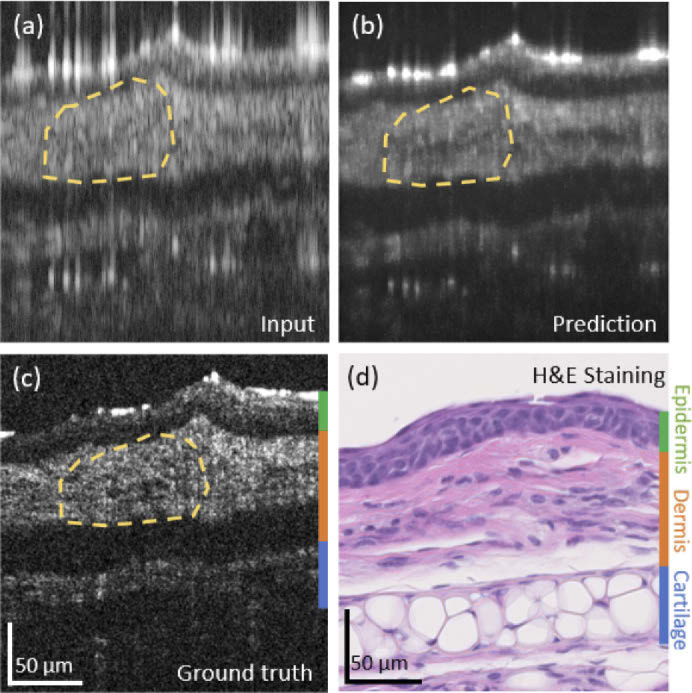
Results of the ex-vivo mouse ear imaging. (a) Input B-scan data. (b) The prediction B-scan data. (c) The ground truth B-scan data. (d) The H&E stained histological micrograph. The layers in the mouse ear are marked by color bands in the histology and OCT data (green = Epidermis, orange = Dermis, blue = Cartilage)

The ex-vivo mouse ear imaging and prediction results are shown in Fig. 6. Figure 6(a)-(c) shows the input, the prediction and the ground truth B-scan data, respectively. In Fig. 6(d) the corresponding H&E stained histology image is shown. From the histology images it can be seen that corresponding morphological features which could be resolved with the full spectrum, Fig. 6(c) were not visible anymore in the discontinuous source generated B-scan data, Fig. 6(a). However the cGAN was able to reconstruct these features and additionally remove side-lobe artifacts, see Fig. 6(b). Especially in the region of the dermis, the cGAN was able to reconstruct fine anatomical features which were not visible in the data reconstructed by the discontinuous source, see orange dashed line in Fig. 6(a)-(c), respectively. The layers found in the mouse ear are marked in the histological micrograph and the OCT cross-section by color bands, where green indicates the epidermis, orange the dermis and blue the cartilage.

### 3.4 Quantitative evaluation

**Fig. 7.**
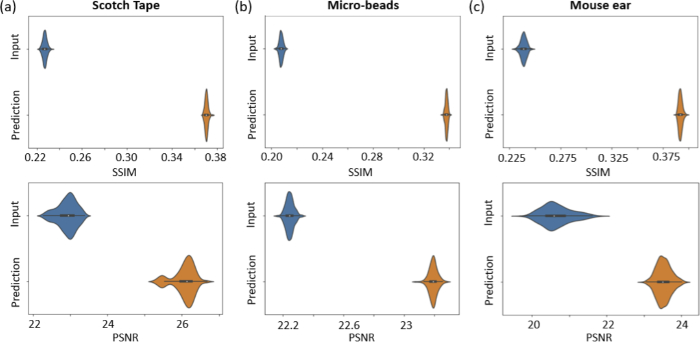
Quantitative evaluation of the cGAN. (a) The SSIM and the PSNR evaluation for the scotch tape data. (b) The SSIM and the PSNR evaluation for the micro-bead data. (c) The SSIM and the PSNR evaluation for the mouse ear data. For each evaluation the ground truth data were evaluated against the predicted (orange) and the input data (blue).

The SSIM and the PSNR as well as the Fréchet Inception Distance were evaluated for the input and predicted data versus the ground truth data. Table 1 shows the mean and standard deviation values for the SSIM, the PSNR values and the Fréchet Inception Distance (FID), for the input (I) and the predicted images (P) versus the ground truth data respectively. Figure 7 shows violin plots of the SSIM and PSNR comparing the generated and ground truth (using the full spectrum) data (orange) versus the input (using the discontinuous spectrum) and ground truth data (blue) for the scotch tape (a), the micro-beads (b) and the ex-vivo mouse ear tissue (c), respectively. The improvement in SSIM and PSNR for all data types can be observed.

**Table 1.** The mean and standard deviation (mean ± std) values for the structural similarity index (SSIM) and the peak signal-to-noise ratio (PSNR) as well as the Fréchet Inception Distance (FID) values for the input (I) and the predicted (P) images versus the ground truth data for the scotch tape, the mirco-beads and the mouse ear.

Metric	Scotch Tape	Micro-bead	Mouse Ear
SSIM(I)	0.23 ± 0.002	0.21 ± 0.001	0.24 ± 0.003
PSNR(I)	22.91 ± 0.25	22.24 ± 0.03	20.65 ± 0.39
FID(I)	319.87	304.61	331.48

SSIM(P)	0.37 ± 0.001	0.34 ± 0.001	0.39 ± 0.002
PSNR(P)	26.06 ± 0.29	23.19 ± 0.003	23.52 ± 0.19
FID(P)	75.13	57.11	77.45

## 4. Discussion

To the best of our knowledge, for the first time, a cGAN was utilized to reconstruct the depth information in OCT data generated by a discontinuous source. The presented cGAN retrieved high resolution OCT data with improved axial resolution and decreased image artifacts from data acquired with a discontinuous source, see Fig. 5 and Fig. 6. The cGAN utilized was inspired by the Pix2Pix Network from Isola et al. [45] and has been adapted to input phase and amplitude of the complex OCT data and furthermore an improved loss function was integrated as described in the method section. In deep learning for image reconstruction the question how data are reconstructed is still an open research field. The success of deep learning is said to be attributed to learning powerful representations that are not yet fully understood [54]. We believe, that in the reconstruction process the network learns the different features based on both the vertical and horizontal information provided in the tomograms. Note, that OCT as a coherent imaging technique exhibits speckle modulating the actual sample structure, and being basically a three dimensional phenomenon.

**Fig. 8.**
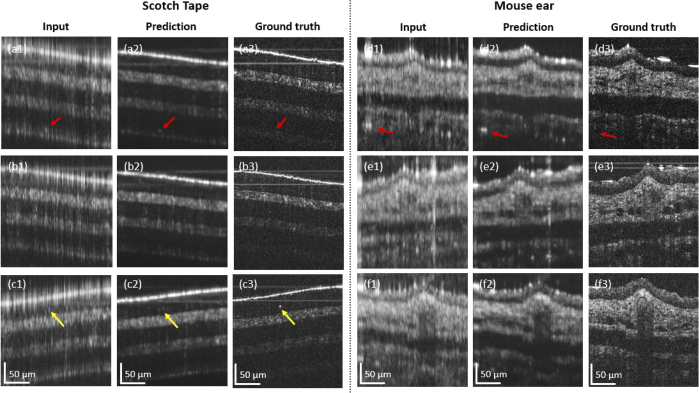
Input, prediction and ground truth results for the scotch tape and the mouse ear tissue. (a1) - (c1) Input, (a2) - (c2) prediction and (a3) - (c3) ground truth data, respectively for the scotch tape imaging. (d1) - (f1) Input, (d2) - (f2) prediction and (d3) - (f3) ground truth data, respectively for the mouse ear imaging.

When comparing the results achieved for the phantoms (Fig. 5 and Fig. 8(a)-(c)) and the ex-vivo mouse ear tissue (Fig. 6 and Fig. 8(d)-(f)), the improvement values observed from SSIM and the PSNR as well as the Fréchet Inception Distance values were rather similar, see Table 1 and Fig. 7. The Fréchet Inception Distance compares the distribution of the generated images with the distribution of the ground truth data [47,48,55], the lower the value the better the similarity in two images is. The PSNR calculates the peak signal-to-noise ratio between the two images in decibels. This ratio is used to measure the quality of the original and the predicted data and the higher this value gets, the better is the quality of the reconstructed image [50]. For our cGAN normalized images with double precision were used, where PSNR values between 20 to 30 dB are generally acceptable [56]. Unlike PSNR, SSIM is based on visible structures in the image [49]. Also for the SSIM higher values, closer to 1, where 1 would indicate an identical image, show higher structural similarity in the images. When comparing the images visually, it seems that the prediction in the phantom data worked still better compared to the mouse ear data. One reason could be, that the mouse ear was stored in formalin and during the continuous imaging process outside the liquid, the tissue starts to dry and shrink and therefore small changes were introduced. These changes however, are difficult to compensate for in the cGAN. That is why for the final training process only synthetically generated discontinuous data from the mouse ear measurements were used. In the future, different hyper-parameters for the cGAN will be tested and especially different loss functions can improve the performance of the cGAN [57]. Another approach, which could be interesting for the future, would be to use a three-dimensional cGAN to predict whole volumes [58]. Nevertheless using the presented cGAN an improvement of axial resolution by a factor of 5 (Fig. 5(c1) and (c2)) could be achieved in combination with decreasing the image artefact introduced by the discontinuous spectrum.

In the scotch tape and the ex-vivo mouse ear imaging results (Fig. 8) it can be observed that the noise in the background gets suppressed by the cGAN and consequently the signal to noise ratio is improved. Especially, in regions of low SNR features arising from side lobe artefacts, were less successfully removed by the cGAN, indicated by red arrows in Fig. 8(a1)-(a3) and (d1)-(d3), respectively. Previous work reported similar observations when reconstructing the resolution of OCT images based on a cGAN [40]. The cGAN blurs the original image in the lateral direction, however for the scotch tape and the micro-beads this effect is not as severe as for the mouse ear tissue. The lateral blurring leads to a suppression of some fine details, highlighted by the yellow arrows in Fig. 8(c1) - (c3), respectively. To improve the sharpness of the images the weights of the loss function could further be investigated. When comparing the features in the ground truth and predicted results for the mouse ear tissue (Fig. 6 and Fig. 8), some differences can be observed. These discrepancies are the result of three factors, the blur, the resolution difference by a factor of four and the slight misalignment between ground truth and input data, as described in the last paragraph. When comparing the input and the ground truth for the scotch tape data (Fig. 5(a1) and (a3) and Fig. 8(a) and (b)) it seems that the imaging depth is increased, one can observe a structure at the bottom of Fig. 5(a1) and Fig. 8(a), respectively. This structures correspond to an artifact introduced by the sidelopes, which are generated by using a discontinuous source.

The effect of the sample on the point-spread-function and in following on the sidelobe artefacts introduced by the discontinuous spectrum, depends on the scattering signal among other factors such as absorption or polarization. By using the micro-beads, the scotch tape, and a biological tissue we showed that our cGAN approach can be used to reconstruct data of a variety of highly and less scattering samples. As future work the effect of the backscattered spectrum could be studied in a controlled manner by trying to reconstruct OCT data from different particles sizes which have been shown to have a strong impact on this aspect [59].

**Fig. 9.**
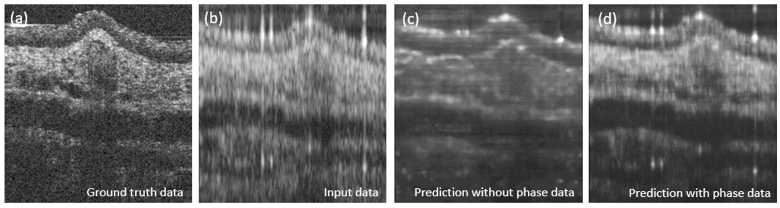
Prediction results for the mouse ear tissue without and with using the phase data for the cGAN. (a) Ground truth image acquired with the full visible spectrum. (b) Input data acquired with the discontinuous source. (c) Prediction based only on the amplitude and (d) on amplitude and phase data.

**Fig. 10.**
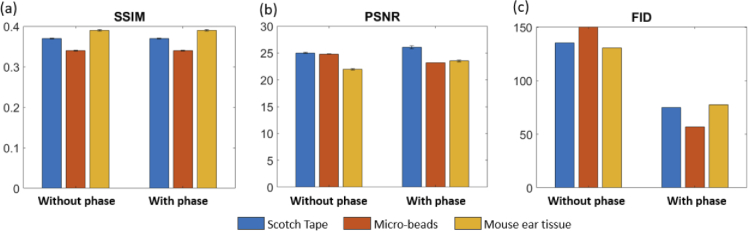
Bar plots of the SSIM, the PSNR and the FID scores for the cGAN prediction results without and with using the phase data, respectively. The error bars represent the standard-deviations.

One novelty of the presented approach was the usage of phase and amplitude data to train our cGAN. Therefore, the importance of using both data as an input for our network was investigated. When training the same model without utilizing the phase data as an additional input, a decrease in the image quality was observed see Fig. 9. Figure 9 shows the predicted results on the test data set for the mouse ear tissue using the cGAN without (c) and with (d) the phase data, respectively. Fewer details and a decreased SNR could be observed. We believe that the main factor, why these additional input data support the training process, is that the phase is preserved within the signal region or to be more precise within each speckle both in lateral and depth direction. Thereby it helps the network to better differentiate between signal and noise regions. This improvement was also quantitatively manifested in significantly aggravated FID scores (see Fig. 10(c)). Figure 2 shows bar plots of the PSNR, SSIM and FID values when traing the CGAN without the phase data as an additional input.

The PSNR and SSIM values didn’t show any significant changes ((see Fig. 10(a) and 10(b)), nonetheless from an visual inspection a clear improvement could be observed. Literature suggests that even tough these scores can measure distortions between the generated and the ground truth images, a higher value not necessarily always guarantees a better quality of the reconstructed images. [60] Additionally, literature has shown, that low SSIM and PSNR in cGAN based OCT image reconstruction arise from the different generated speckle patterns [40]. In the future a comprehensive subjective evaluation, using for example a mean opinion score, could be conducted to get a deeper insight into the improvements achieved. Nevertheless, we believe, that the phase data, helped the network to focus on regions including tissue structures and therefore improved the cGAN performance.

The total output power in the visible wavelength (350-850 nm) region of the NKT supercontinuum source was 600 mW. For laser safety reasons for the operator and to avoid photochemical and thermal changes in the tissue during imaging, the power was reduced to 0.8 mW at the sample. The discontinuous EXALOS source can provide 5 mW output power per color channel. For the presented results the total output power was also reduced to have 0.8 mW at the sample to generate comparable input data for the cGAN. The input power of the different color channels was scaled in a way that it follows the shape of the original visible light spectrum. However, by exploring the full power range of this EXALOS source, red-green-blue (RGB) based OCT imaging or even other spectroscopic applications could be investigated [61–63]. Further, the source could be used to perform simultaneously OCT and fluorescence imaging, to gain additional tissue specific contrast [64,65].

Current state-of-the-art high-resolution OCT systems push the limits of commercially available optics by requiring consistent optical performance over a large spectral bandwidth. This increases both the cost and complexity of these systems. In this work, we overcome this limitation by training a cGAN. The big advantage of the presented work, in comparisons to numerical solutions to improve the image quality, when using discontinuous spectral data [21] or the axial resolution [66], is that the cGAN is completely independent from any required pre-knowledge about the spectrum or the setup. As soon as the cGAN is trained, the prediction of new data sets can be performed independently from the supercontinuum data in real time. Only the processed low resolution image data were used as an input, to predict the corresponding high resolution images. The EXALOS RGB in comparison to the supercontinuum NKT source is compact, of lower cost and in general SLEDs have a lower relative intensity noise [13]. As a next step, data acquired with discontinuous sources having different wavelength peaks, could be used to train and test our cGAN, increasing the flexibility of the network and therefore explore the possibility of a more universal application of the presented approach. In this context the influence of the used spectral with and location of the peaks will further be investigated, to explore the possibilities of our cGAN network.

**Fig. 11.**
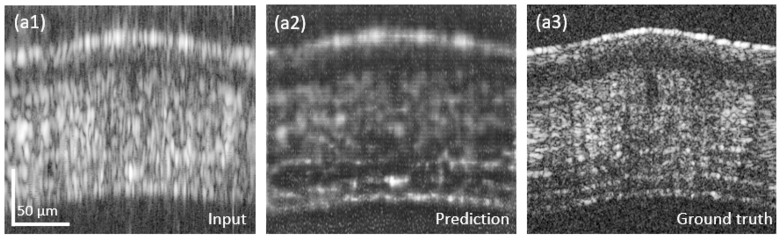
Input, prediction and ground truth results for the mouse cornea imaging.

Furthermore, the presented results so far, were obtained from healthy mouse ear tissue. However, all predicted images presented in this manuscript were obtained from unknown data to our cGAN. For a first evaluation of our approach on a tissue morphology that the network was not trained on, we additionally imaged an ex-vivo mouse cornea sample. Figure 11(a1) - (a3) shows the input, the predicted and the ground truth B-scan data, respectively. As described by another research group, which used a cGAN to improve the image quality of OCT data [40], the network was not able to generate high-quality results, as obtained in the mouse ear sample. Although the cGAN was able to improve the resolution and reduce artefact introduced by the discontinuous spectrum. As a next step tissue types showing abnormalities, such as tumors will be investigated and used for the training and prediction process. A thorough study of the applicability of our network to other tissue types will be conducted. Cancerous tissue can show different features in OCT images; therefore, the prediction of such images would be of high interest to further test and validate our approach.

In conclusion, the results are a promising first step to reconstruct the depth resolution of OCT images generated by a discontinuous optical spectrum using a machine learning approach. Therefore, the cGAN presented in this article opens the horizon for multiple other applications in the field of OCT using discontinuous light sources.

## 5. Conclusion

In this work, a cGAN was utilized to reconstruct high-resolution and artefact-reduced images generated by a discontinuous source. The network was trained to recover the depth resolution of OCT images generated by an EXALOS SLD laser with a discontinuous spectrum in the visible wavelength region. The cGAN learned the relation between low- and high-resolution data and consequently how to obtain images reconstructed by the full visible spectrum from the discontinuous one. As an input for the network, the phase and the amplitude of the complex OCT data were utilized. The reconstruction performance of the framework proposed was tested using three different data types: a layered phantom, micro-beads and ex-vivo mouse ear tissue. The results presented are significant, as our approach showed that using a cGAN an improved axial resolution and improved image quality can be achieved approaching the original high-resolution data. Therefore, the presented work opens the horizon for various other applications in the field of OCT using discontinuous light sources.

## Data Availability

The network presented in this article is available in Ref. [67]. Other data underlying the results presented in this paper are not publicly available at this time but may be obtained from the authors upon reasonable request.
